# Reentrant Phenomenon in Barium Titanate Zirconate‐Based Relaxor Ferroelectrics

**DOI:** 10.1002/smll.202501914

**Published:** 2025-07-15

**Authors:** Eva Kröll, Boriana Mihailova, Vadzim Haronin, Jūras Banys, Doru C. Lupascu, Vladimir V. Shvartsman

**Affiliations:** ^1^ Institute for Materials Science and Center for Nanointegration Duisburg‐Essen (CENIDE) University of Duisburg‐Essen 45141 Essen Germany; ^2^ Department of Earth System Sciences Universität Hamburg 20146 Hamburg Germany; ^3^ Institute of Applied Electrodynamics and Telecommunications (IAET) Vilnius University Vilnius LT‐10257 Lithuania

**Keywords:** barium titanate zirconate, Raman spectroscopy, Reentrant transition, relaxor ferroelectric, solid solution

## Abstract

Reentrant phenomena are rare occurrences in relaxor ferroelectrics, and their underlying mechanisms are still unknown. In this paper, we report on the reentrant relaxor behavior in the perovskite system (1 − *x*)Ba(Ti_0.85_Zr_0.15_)O_3_‐*x*Bi(Zn_2/3_Nb_1/3_)O_3_ with *x*  = 0, 0.015, 0.02, 0.04, 0.06, and 0.15. For all compositions, the temperature dependences of the dielectric permittivity exhibit characteristic reentrant features: a frequency‐independent maximum ≈ 340 K (*T*_m_) associated with the ferroelectric phase transition, and a frequency‐dependent anomaly (*T*_R_) at lower temperatures indicating relaxor behavior. Raman spectroscopy shows that the correlation length of the B‐cation displacements is suppressed by doping resulting in a cross‐over from macroscopic to mesoscopic polar order. On the other hand, correlated antiferrodistortive rotations of the oxygen octahedra give rise to nanometer size clusters of antipolar shifted A‐site cations distributed within the polar matrix. Slowing down of the dynamics of these clusters leads to low‐temperature dielectric anomaly and reentrant relaxor behavior. Although competition between polar modes and antiferrodistortive modes is a common feature of perovskite ferroelectrics, it is shown that it can be the origin of the reentrant relaxor behavior.

## Graphical abstract

This study explores reentrant relaxor behavior in (1 − *x*)Ba(Ti_0.85_Zr_0.15_)O_3_‐*x*Bi(Zn_2/3_Nb_1/3_)O_3_ (BTZr‐BZNb). Substituting with BZNb disrupts long‐range polar order, creating nanometer‐scale “antipolar clusters” within a polar matrix. The competition between polar modes and antiferrodistortive rotations leads to low‐temperature relaxor anomalies. Increased doping weakens cluster interactions, intensifying relaxor behavior and transitioning to weakly‐coupled relaxor behavior.

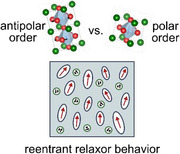

## 1 Introduction

Upon decreasing temperature, thermodynamic systems usually transform from a disordered high symmetry phase to a more ordered phase with lower symmetry. However, in some instances an unusual phase sequence is observed when after this “normal” transition the system reenters a disordered phase upon further cooling. In this situation, the low‐temperature state, which is not exactly the same as the high‐temperature disordered state, is called a reentrant phase. Reentrant phenomena have been found in various systems, including binary liquid mixtures,^[^ ^1^ ^]^ liquid crystals,^[^ ^2^ ^]^ superconductors,^[^ ^3^ ^]^ spin glasses,^[^ ^4^ ^]^ and relaxor ferroelectrics.^[^ ^5^ ^]^ Temperature‐induced phase transitions are generally related to the minimization of the free energy *F*, which is defined as: *F*  =  *U* − *TS*. Usually, the ordered state has lower internal energy, *U*, than the disordered state. At high temperatures, the disorder prevails due to the enlarged contribution of the entropy *S*. The transition from a disordered to an ordered state occurs when the increase of the –*TS* term is counterbalanced by the decrease of internal energy *U*. However, at lower temperatures when the reentrant transition takes place, the vanishing/reduction of the order parameter is accompanied by much smaller decrease in− *TS* than the corresponding increase of *U*. Therefore, the reentrant transition probably reflects hidden mechanisms that keep the free energy, *F*, low.^[^ ^6^ ^]^

These mechanisms have been intensively studied in reentrant spin‐glasses. In these materials, a ferromagnetic (FM) or an antiferromagnetic (AFM) phase is diluted with magnetic or non‐magnetic components. In a specific concentration range, a reentrant spin‐glass phase appears at low temperatures in addition to the (anti)ferromagnetic to paramagnetic phase transition at higher temperatures. Based on the studies of reentrant spin‐glasses, two different theories were developed to explain the microscopic origin behind the reentrant phenomenon: the transverse (*xy*) freezing model,^[^ ^4^ ^]^ and the random‐field model.^[^ ^7^, ^8^ ^]^

The transverse freezing model suggests that below the Curie point *T*_C_, the magnetic moments become ordered along the *z* axis but stay dynamically disordered in the *xy* plane. Upon cooling to the reentrant temperature *T*_R_, all components of the magnetic moments become static, introducing orientation disorder within the *xy* plane, which perturbs the already established order along the *z* direction.

In contrast, the random‐field model is based on the percolation theory, where two coexisting phases are present below *T*_C_. One phase consists of finite dynamic FM clusters which are distributed within an infinite static FM matrix that represents the other phase. Below *T*_R_, all random spins freeze and form an infinite FM cluster with static random fields.

Analogues to reentrant spin‐glasses, reentrant relaxor ferroelectrics (RRF) were discovered in BaTiO_3_‐BiScO_3_ solid solutions,^[^ ^5^, ^9^ ^]^ (Ba_0.925_Bi_0.05_)(Ti_1‐x_Sn_x_)O_3_,^[^ ^10^ ^]^ PbCo_1/3_Nb_2/3_O_3_ ceramics,^[^ ^11^ ^]^ and in some tungsten bronze systems.^[^ ^12^, ^13^, ^14^ ^]^ The generic feature of the reentrant phase transition is a pronounced frequency dependent anomaly in the temperature dependence of the dielectric permittivity at temperatures much below the frequency independent peak of *ε*(T) associated with the ferroelectric‐paraelectric phase transition. Similar behavior was recently observed also in (1 − *x*)Pb(Mg_1/3_Nb_2/3_)O_3_‐*x*PbTiO_3_ (PMN‐PT) with *x*  = 0.15, and 0.30.^[^ ^10^ ^]^ The proposed model considers these materials as composites of polar nanoregions (PNRs) and long‐range ferroelectric domains and is similar to the random‐field model of the reentrant state. In some cases a decrease in the remanent polarization has been reported below the reentrant transition temperature, which was interpreted as an indication of destabilization of the long‐range ferroelectric order.^[^ ^13^ ^]^ However, a similar trend can be caused by an increase in the coercive field upon cooling leading to incomplete polarizing of the sample.

The origin of the reentrant relaxor state is still a matter of discussion. A complex approach addressing the reentrant phenomena by different experimental techniques may shed a light on its mechanisms. In this paper, we report the reentrant relaxor behavior in a novel system of solid solutions of (1 − *x*)Ba(Ti_0.85_Zr_0.15_)O_3_‐*x*Bi(Zn_2/3_Nb_1/3_)O_3_ (BTZr‐*x*BZNb) with *x*  = 0 – 0.15. For convenience, the samples will be referred to as BTZr and *x*BZNb, where *x* corresponds to the mole fraction. The reentrant relaxor behavior was detected by low‐temperature dielectric measurements. It is manifested by an anomaly with distinct frequency dispersion at temperatures well below that the frequency‐independent maximum on *ε*(*T*) corresponding to the diffuse ferroelectric phase transition. To understand the mechanism behind the reentrant phase, the compositions with *x*  = 0, 0.02, 0.06, and 0.15 were further studied by in situ temperature‐dependent Raman spectroscopy.

## 2 Results

### 2.1 Dielectric Spectroscopy

**Figure 1:**
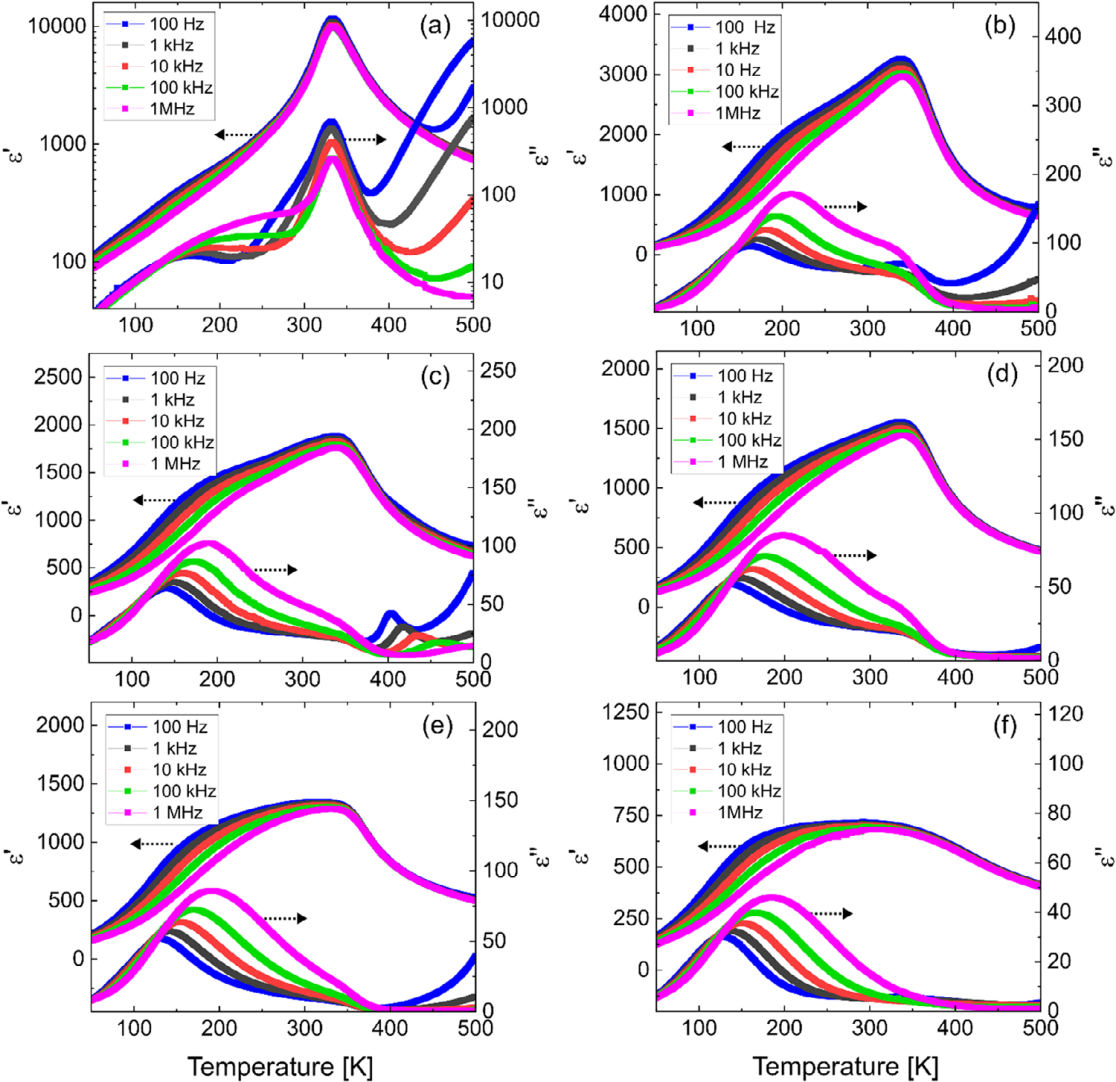
Temperature‐dependent real and imaginary parts of the dielectric permittivity for the BTZr‐BZNb samples with *x* = 0 a), 0.015 b), 0.02 c), 0.04 d), 0.06 e), and 0.15 f) measured in the frequency range of 100 Hz‐1 MHz.

Ceramic samples of (1‐*x*)Ba(Ti_0.85_Zr_0.15_)O_3_‐*x*Bi(Zn_2/3_Nb_1/3_)O_3_ (BTZr‐*x*BZNb) with *x* = 0 – 0.15 were synthesized using the solid‐state method. The details of the sample preparation, results of the X‐ray diffraction (XRD), high–temperature dielectric and polarization measurements can be found in Ref.[15] For convenience, the samples will be referred to as BTZr and *x*BZNb, where *x* corresponds to the mole fraction of Bi(Zn_2/3_Nb_1/3_)O_3_. **Figure** **1** shows the temperature‐dependent real and imaginary parts of the dielectric permittivity of the BTZr‐*x*BZNb samples with ***x *** = 0, 0.015, 0.02, 0.04, 0.06, and 0.15. The base composition BTZr exhibits a broad frequency independent maximum at 334 K (*T*_m_), which is attributed to a diffuse phase transition between paraelectric and ferroelectric phase.^[^ ^16^ ^]^ In addition, a second anomaly with distinct frequency dispersion occurs at ≈226 K (*T*_R_), noticeable as a shoulder in the real part of dielectric permittivity, *ε´*(*T*), and as a peak in the imaginary part, *ε´´*(*T*), when plotted on a logarithmic scale. Interestingly, this anomaly has not been previously observed for BTZr, but similar behavior was recently reported for Ba(Ti_0.85_Sn_0.15_)O_3_ (BTSn).^[^ ^17^ ^]^ As the amount of BZNb increases, the peak permittivity drops significantly to lower values, and the second anomaly becomes more pronounced and is distinct also on a linear scale. **Table** **1** lists *T*_m_ and *T*_R_ at 100 kHz for all compositions, where *T_R_* was estimated from the position of the maximum of the imaginary part of dielectric permittivity. Both characteristic temperatures initially increase with the addition of BZNb but decrease with further increasing amounts of BZNb (for *x* ≥ 0.04). For the compositions with *x* = 0.06 and 0.15, the real part of the dielectric permittivity at *T_m_* becomes comparable in magnitude with the dielectric permittivity at *T_R_*. As a result, the temperature dependences of *ε´*(*T*) attain a form of plateau between *T_R_* and *T_m_* with a strong frequency dispersion at the low temperature side. The anomaly of the imaginary part (a maximum for BTZr and a “step” for *x* ≥ 0.02) is suppressed for increasing amount of BZNb and vanishes almost completely for the samples with ***x *** = 0.15. However, the dielectric data do not clearly indicate whether the two transitions overlap, or if the ferroelectric‐paraelectric phase transition has completely vanished for this composition.

**Table 1:** Results of the Vogel‐Fulcher fit for the imaginary part of the second dielectric anomaly and the Burns temperature *T*_B_.

*x*	*T_m_* [K] (100 kHz)	*T_R_* [K] (100 kHz)	*T*_B_ [K] (100 kHz)	*E*_a_ [eV]	*T*_f_ [K]	*f*_0_ [Hz]
0	335	226	376	0.09	96	5.9 × 10^8^
0.015	340	193	385	0.10	106	4.8 × 10^10^
0.02	341	175	378	0.10	82	4.8 × 10^10^
0.04	334	176	375	0.24	30	1.4 × 10^13^
0.06	326	170	371	0.22	31	1.1 × 10^13^
0.15	295	167	350	0.21	25	5.3 × 10^12^

One of the features of the diffuse phase transition is a deviation of the *ε*(*T*) dependences from the Curie‐Weiss law characteristic of canonical ferroelectrics. This deviation is considered to be a manifestation of polar nanoregions (PNRs) appearing below the Burns temperature, *T_B_*.^[^ ^18^ ^]^ One of the ways to determine the Burns temperature is plotting the reciprocal permittivity as a function of temperature, then *T*_B_ corresponds to the point where the curve deviates from linearity^[^ ^10^ ^]^ (see Figure S1, Supporting Information). Using this method, the Burns temperature for BTZr was estimated to be 376 K. With the addition of BZNb, *T*_B_ first increases slightly, but then decreases with a further increase in BZNb (see Table 1).

Appearance of a frequency dependent dielectric anomaly at temperatures below the global ferroelectric‐paraelectric phase transition is considered as an indicator of the reentrant relaxor transition.^[^ ^6^ ^]^ This transition is associated with the critical slowing down (freezing) of dynamic polar entities, PNRs. Usually, the characteristic parameters describing the freezing of PNRs are obtained by fitting the position of the frequency dependent peak of dielectric permittivity *T_R_* (the imaginary part in our case) using the empirical Vogel‐Fulcher law:

(1) $$f\;=f_{0}\exp\left[\frac{-E_{a}}{k\left(T_{R}-T_{f}\right)}\right]$$

where *f* is the frequency, *f*_0_ is the attempt frequency, *E_a_* is the activation energy, and *T_f_* is the freezing temperature.

The best fit parameters are listed in Table 1. It can be seen that samples with ***x*** ≥ 0.04 show a significant increase in both *E*_a_ and *f*_0_, while the freezing temperature drops from 80 – 100 K to 20–30 K.

The relaxor behavior of the samples with *x* ≥ 0.02 at room temperature is confirmed by the slim shape of the polarization hysteresis loops (Figure S2, Supporting Information).

### 2.2 Raman Spectroscopy

**Figure 2:**
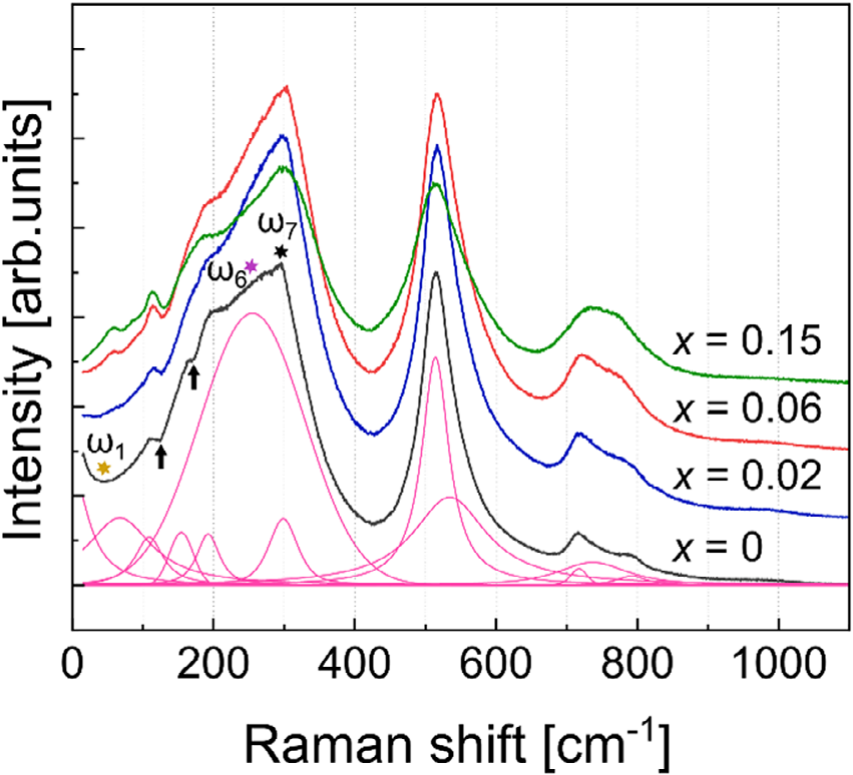
Raman spectra of the parallel‐polarized BTZr‐*x*BZNb samples with *x* = 0, 0.02, 0.06, and 0.15 measured at 300 K. The asterisks mark the three peaks, ω_1_, ω_6_, and ω_7_, whose temperature dependences are discussed in detail. The arrows mark the interference dips at 177 and 127 cm^−1^. The spectra are offset vertically for clarity. The pink curves display the fitting model of the BTZr sample.

Raman spectroscopy was used to further study the structural transformations in the BTZr‐xBZNb solid solution and to clarify the atomistic mechanism behind the reentrant behavior. In situ measurements were performed in the spectral range 15–1215 cm^−1^ on cooling from 700 to 100 K. Experiments before and after the temperature run confirmed the reversibility of all observed changes. **Figure** **2** shows the parallel polarized Raman spectra of BTZr‐*x*BZNb samples with x = 0, 0.02, 0.06, and 0.15 at 300 K (see also Figure S3, Supporting Information). Several distinct features can be observed with increasing amount of BZNb.

The interference dip near 177 cm^−1^ vanishes upon the addition of BZNb, while the dip at 123 cm^−1^ is enhanced. The former results from coupling between the polar A_1_(TO1) and A_1_(TO2) phonon modes in the BaTiO_3_ matrix^[^ ^19^ ^]^ and it is typical of BaTiO_3_‐based ceramics with developed long‐range ferroelectric order.^[^ ^20^, ^21^ ^]^ Thus, its disappearance for *x* > 0 indicates a decrease in the correlation length of coupled polar entities in the BTZr‐*x*BZNb solid solution. The dip near 123 cm^−1^ is attributed to substitution disorder at the B‐site,^[^ ^20^, ^21^ ^]^ which certainly is enhanced with the increase in *x*.

Substitution B‐site disorder in BTZr‐*x*BZNb is also responsible for the enhanced Raman signal near 780 cm^−1^, similar to BaTi_1‐x_Zr_x_O_3_ ceramics.^[^ ^20^, ^22^ ^]^ The co‐substitution of Bi for Ba at the A‐site and (Zn_2/3_Nb_1/3_) for (Ti_0.85_Zr_0.15_) at the B‐site also leads to the decrease of the central‐peak (CP) tail observed below 40 cm^−1^ for BTZr, an enhancement of the Raman scattering near 55 cm^−1^ (labelled with ω_1_ in Figure 2), which consequently becomes better resolved, and an increased intensity ratio between the Raman peaks near 305 and 270 cm^−1^ (ω_7_ and ω_6_ in Figure 2, respectively).

The peak at ω_6_ ∼270 cm^−1^ is associated with vibrations of off‐centered B‐site cations,^[^ ^23^, ^24^ ^]^ whereas the peak at ω_7_ ∼305 cm^−1^ stems from the silent T_2u_ mode of the aristotype perovskite structure and can be considered as BO_6_ tilting in a doubled perovskite structure.^[^ ^23^, ^25^, ^26^ ^]^

According to first‐principles calculations on BaTi_1‐x_Zr_x_O_3_ with *x* = 0.26, the local strains in the oxygen sub‐lattice induced by the homovalent B‐site substitution results in subtle octahedral tilting as well as in off‐centered shifts of the A‐site cations (≈0.06 Å) with respect to their surrounding oxygen atoms.^[^ ^27^ ^]^ The same local structural distortions should be expected in BaTi_1‐x_Zr_x_O_3_ with *x* = 0.15 studied here. On the other hand, density‐functional theory modelling of the phonon density of states for a hypothetical BaZrO_3_ phase comprising antiphase BO_6_ tilts indicates that the low‐energy phonons are dominated by A‐site cation vibrations.^[^ ^28^ ^]^ Calculated partial phonon densities of states of cubic and rhombohedral BaTiO_3_ also reveal the dominance of Ba vibrations below 100 cm^−1^.^[^ ^29^ ^]^ Given that the structure of the base compound BaTi_0.85_Zr_0.15_O_3_ is pseudocubic/rhombohedral,^[^ ^15^, ^20^ ^]^ we attribute the Raman scattering near 55 cm^−1^ in the spectra of BTZr‐*x*BZNb to off‐centered A‐site cations in a double perovskite structural entity associated with antiphase octahedral tilting. Such a peak assignment correlates with the fact that the incorporation of A‐site Bi^3+^ cations, which tend to off‐center, enhances the Raman signal at ω_1_. It should be underlined that dynamic fluctuations involving BO_6_ tilts with a correlation length of 2–3 nm over a picosecond time scale have been demonstrated to exist in BaZrO_3_.^[^ ^30^ ^]^ The full widths at half maximum (FWHMs) of the central feature detected at room temperature for BTZr‐*x*BZNb with *x* = 0, 0.02 and 0.06 is approximately 60–70 cm^−1^, which corresponds to a relaxation time of the order of picoseconds, when a simple Debye relaxation is assumed,^[^ ^31^ ^]^ which is in accordance with the relaxation dynamics in BaZrO_3_. For *x* = 0.15, a CP tail above 15 cm^−1^ was not detected, suggesting slowed‐down dynamics of the structural fluctuations responsible for the frequency dispersion of the dielectric‐permittivity feature near 170 K.

To further clarify the temperature evolution of the local structure, Raman spectra at different temperatures (Figures. S4‐S7) were fitted with pseudo‐Voigt peak‐shape functions to derive the temperature dependences of the phonon wavenumbers ω, FWHMs Γ, and fractional intensities *I_n_*. Below we discuss the temperature behavior of the peaks near ω_1_ ∼50 cm^−1^, ω_6_, ∼270 cm^−1^, and ω_7_ ∼305 cm^−1^ in detail, as these modes are sensitive to the three major local ferroic distortions in the perovskite structure: polar distortions of the AO_12_ cuboctahedra, polar distortions of the BO_6_ octahedra, and BO_6_ tilting, respectively.^[^ ^32^ ^]^

**Figure 3:**
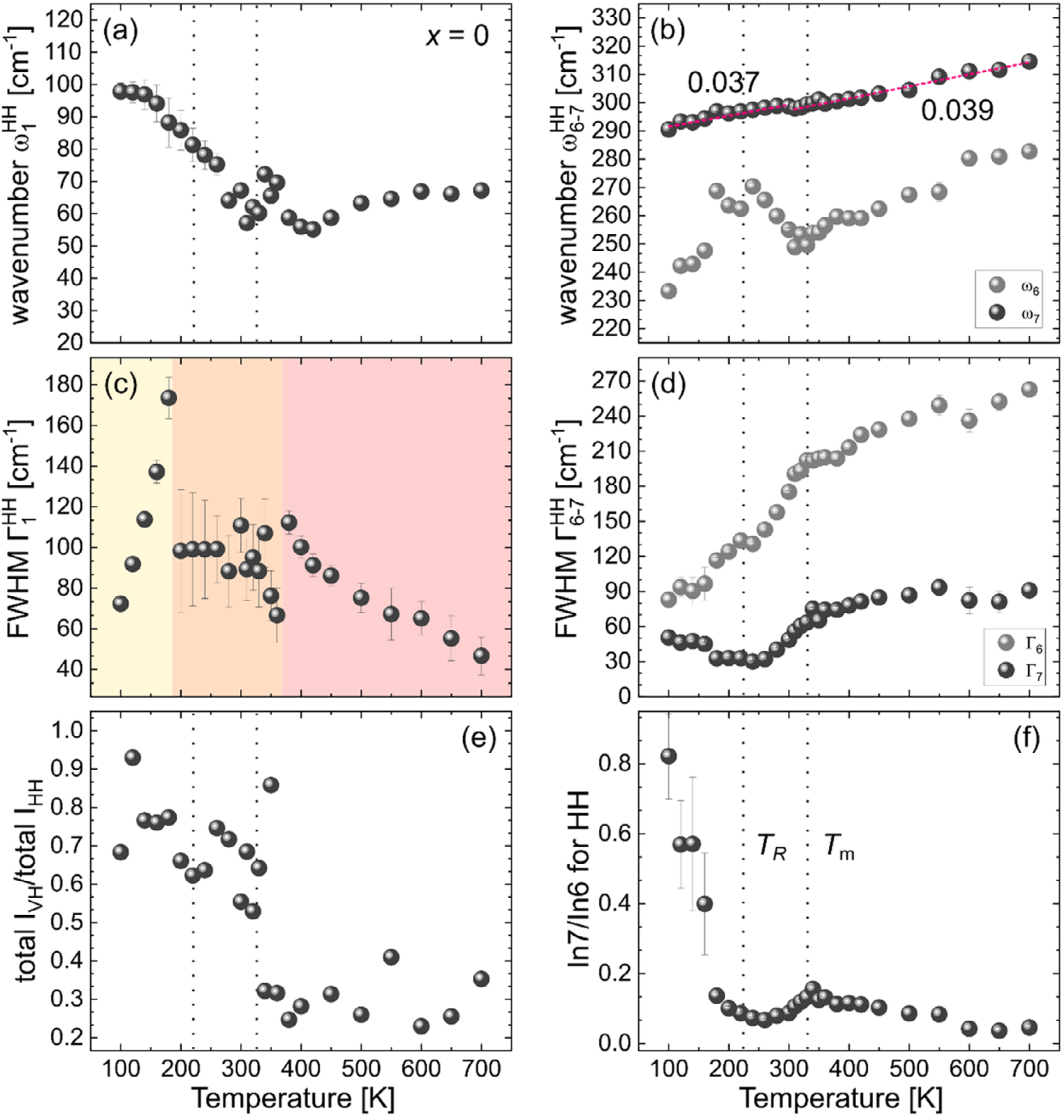
Temperature dependence of the Raman‐peak positions for ω_1_ (a), ω_6_, and ω_7_ (b), their corresponding FWHM (c, d) in the parallel‐polarized (HH) spectra, the intensity ratio of the cross‐polarized (VH) and parallel‐polarized (HH) spectra (e), and the fractional intensity ratio of the peaks ω_7_ and ω_6_ (f) for the BTZr sample. The data points of ω_7_(T) below and above *T*_m_ are linearly fitted and their corresponding slopes are given in plot (b).

**Figure** **3** displays the results of the fittings for the base composition BTZr. To fit the spectrum profile in the temperature range 200–360 K it was necessary to add a CP feature at 0 cm^−1^. Comparison of the anti‐Stokes and Stokes spectral profiles at 300 K also suggests the presence of a CP. However, the use of a CP below 200 K and above 360 K led to large uncertainties in the peak intensities and hence, a 0‐cm^−1^ peak was excluded from the fitting model in the corresponding temperature ranges, based on the criteria of the null‐hypothesis significance testing (see supplementary material in ^[^ ^25^ ^]^). Note that the lower and upper limits of the intermediate temperature region where the CP exists are close to *T_R_* and *T_B_* derived from dielectric permittivity data. Due to the overlap of the central peak and the phonon mode at ω_1_, Γ_1_(*T*) can be divided into three different temperature regions (Figure 3c): in the presence of the CP (200–360 K) Γ_1_(*T*) remains more or less the same within uncertainties, whereas Γ_1_(*T*) increases when approaching 200 K from below and 360 K from above, suggesting coupling of the dynamical flipping of the structure (i.e. CP) with the vibrations of off‐centered A‐site cations. Moreover, ω_1_(*T*) exhibits a plateau‐like minimum approximately between *T*_m_ and *T*_B_ (Figure 3a), while ω_6_(*T*) has a minimum exactly at *T*_m_ (Figure 3b). The former indicates ongoing rearrangements within the A‐site sub‐lattice between *T_B_* and *T*_m_, whereas the latter demonstrates that the dielectric permittivity peak at *T*_m_ originates mainly from the coupling of the local dipoles within the B‐site sub‐lattice. The abrupt step‐wise increase in the depolarization ratio *I*_VH_/*I*_HH_ below *T*_m_ (Figure 3e), which is indicative of the development of ferroelectric long‐range order,^[^ ^32^, ^33^ ^]^ also nicely correlates with the peak permittivity at *T*_m_.

Interestingly, the BO_6_‐tilt mode anomalously softens with decreasing temperature in the entire temperate range 100–700 K and ω_7_(*T*) exhibits only a subtle kink at *T*_m_, marking the border between two different regions that can be fitted linearly with two different slopes $\frac{d\omega }{\mathrm{dT}}$, given in Figure 3b. The FWHM of both the BO_6_‐tilt and off‐centered B‐cation modes, Γ_7_(*T*) and Γ_6_(*T*), have anomalous excess near *T*_m_ (Figure 3d), suggesting that, to a certain extent, these two phonon modes interfere with each other. The second anomaly *T*_R_ of the dielectric data is clearly marked by the beginning of anomalous softening of ω_6_(*T*) below 180 K (see Figure 3b) as well as by a drastic increase in the intensity ratio In_7_/In_6_ (Figure 3f) upon cooling. This suggests that the reentrant relaxor state formed in BTZr below *T*_R_ is related to the development of correlated BO_6_ tilts, which compete with the already existing correlated polar shifts of the B‐site cations and destabilize the coherence between B‐site local dipoles. In other words, a secondary order parameter, representing antiferrodistortive order of tilted BO_6_ octahedra, evolves below *T*_R_ which strongly affects and even overpowers the primary order parameter, representing polar order of distorted BO_6_ octahedra, leading to an increase in the entropy with the temperature decrease.

**Figure 4:**
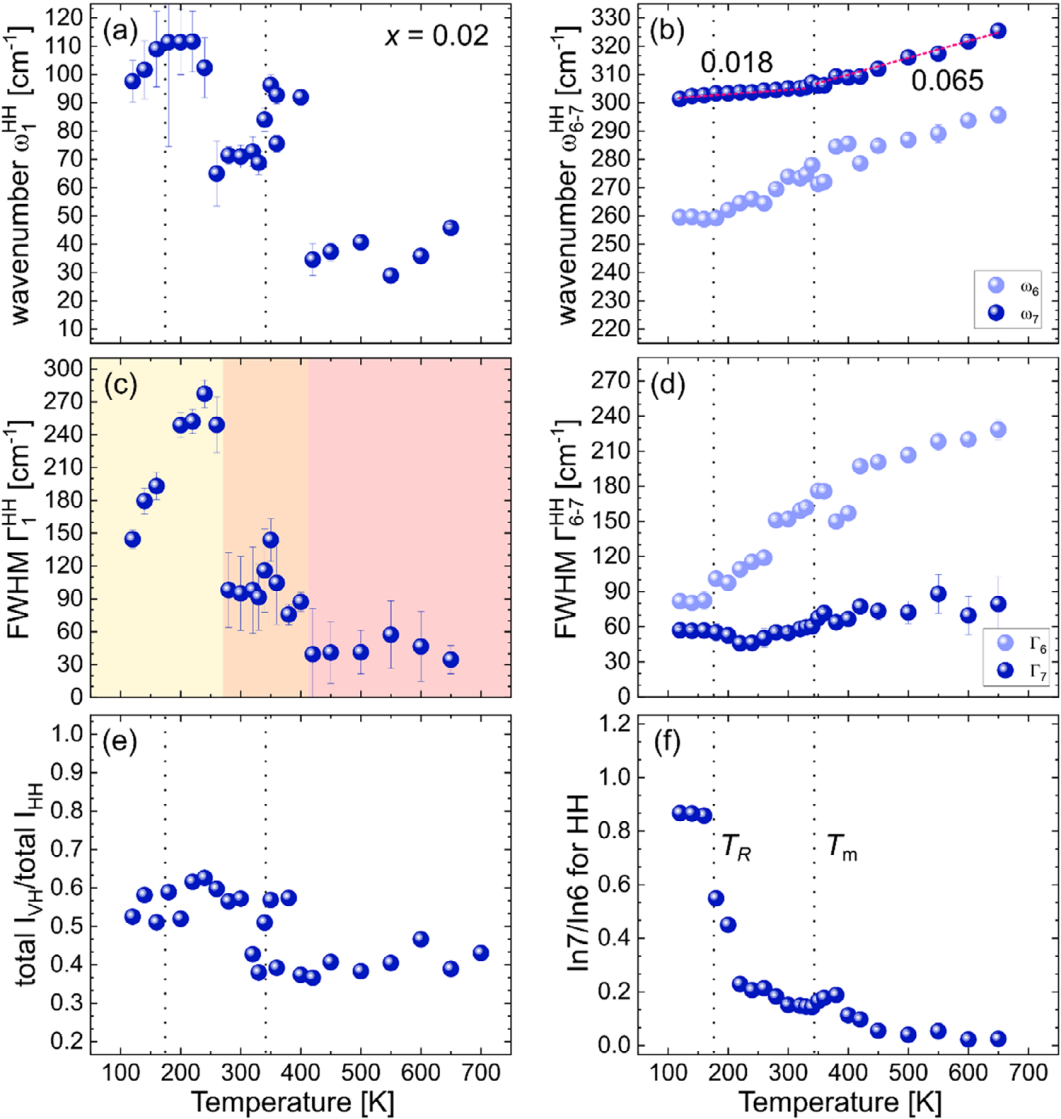
Temperature dependence of the Raman‐peak positions for ω_1_ (a), ω_6_, and ω_7_ (b), their corresponding FWHM (c, d) in the parallel‐polarized (HH) spectra, the intensity ratio of the cross‐polarized (VH) and parallel‐polarized (HH) spectra (e), and the fractional intensity ratio of the peaks ω_7_ and ω_6_ (f) for the BTZr‐BZNb sample with *x* = 0.02. The data points of ω_7_(T) below and above *T*_m_ are linearly fitted and their corresponding slopes are given in the plot (b).

**Figure** **4** shows the fitting results for the 2BZNb sample. Similar to BTZr, the 2BZNb compound exhibits a CP feature between 260–400 K that divides Γ_1_ into three regions (Figure 4c). The phonon mode of ω_1_(T) shows a sudden jump at the upper border of the intermediate region, which is slightly above *T*_B_ and a plateau below *T*_R_. The phonon modes related to off‐centered B‐site cations and BO_6_ tilts show a gradual decrease of ω_6_(T) and ω_7_(T) upon cooling. Like BTZr, a kink at *T*_m_ is observed for ω_7_(T) that marks a distinct change of the slope in the linear regions. The mode of ω_6_(T) also exhibits a kink near *T*_m_ and reaches a plateau‐like minimum at temperatures below *T*_R_ (Figure 4b). The absence of a clear minimum of ω_1_(T) and ω_6_(T) as well as of a kink in Γ_6_(T) at *T*_m_ (Figure 4d) indicates that the addition of BZNb disturbs the correlation length between the off‐centered cations in both the A‐site and B‐site sub‐lattices. The total‐intensity ratio I_VH_/I_HH_ shows an abrupt jump at *T*_m_ (Figure 4e), indicating a development of domains with long‐range ferroelectric order as in the case of BTZr, but the lower value of *I*_VH_/*I*_HH_ below *T*_m_ as compared to that of BTZr suggests a reduction of the correlation length of coherent polar distortions. The softening of the tilt mode below *T*_m_ (Figure 4b) is less pronounced for 2BZNb as compared to BTZr. The ratio of the fractional intensity In7/In6 shows similar behavior to that of BTZr: a slight maximum in the vicinity of *T*_m_ and a strong increase near *T*_R_ (Figure 4f) reaching the same value as in the case of BTZr. This suggests the same mechanism of the formation of the reentrant disordered phase as for BTZr. However, ω_6_(T) remains constant on cooling below *T*_R_ (Figure 4b), instead to anomalously decrease as for BTZ, i.e., the destabilization of the coherent B‐site local dipoles slows down upon the incorporation of BZNb.

**Figure 5:**
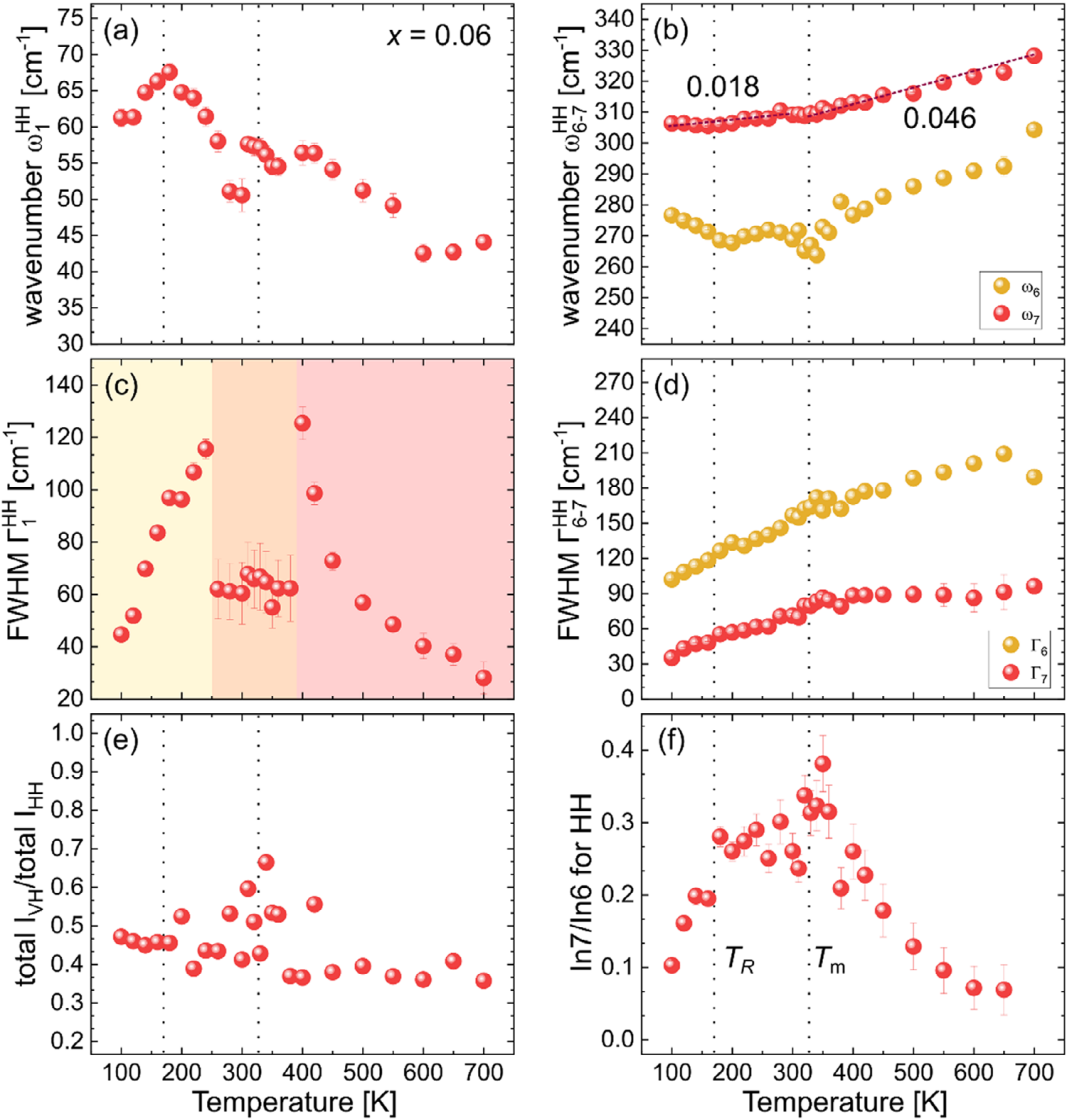
Temperature dependence of the Raman‐peak positions for ω_1_ (a), ω_6_, and ω_7_ (b), their corresponding FWHM (c, d) in the parallel‐polarized (HH) spectra, the intensity ratio of the cross‐polarized (VH) and parallel‐polarized (HH) spectra (e), and the fractional intensity ratio of the peaks ω_7_ and ω_6_ (f) for the BTZ‐BZNb sample with *x* = 0.06. The data points of ω_7_(T) below and above T_m_ are linearly fitted and their corresponding slopes are given in plot (b).

**Figure** **5** shows the results from spectral fittings for the 6BZNb sample. For this compound, a CP feature was necessary to be included in the range 260–380 K. This temperature range defines the borders between the three regions and corresponds to a plateau in Γ_1_(T) (Figure 5c). In contrast to BTZr and 2BZNb, ω_1_(T) clearly decreases upon cooling below *T*_R_ (Figure 5a), while ω_6_(T) exhibits two minima that mirror both dielectric features at *T*_R_ and *T*_m_. Similar to 2BZNb, ω_7_(T) exhibits a kink at *T*_m_ with quantitatively the same slope $\frac{d\omega }{\mathrm{dT}}$ at lower temperatures (Figure 5b). The depolarization ratio *I*_VH_/*I*_HH_ remains almost constant (Figure 5e), without an abrupt jump as in BTZr and 2BZNb, showing only a small maximum near *T*_m_. This suggests the absence of domains with long‐range ferroelectric order. The temperature dependence of the intensity ratio In7/In6 for 6BZNb is also completely different from those of BTZr and 2BZNb (Figure 5f). Rather interestingly, for BZNb‐doping with *x* = 0.06, In7/In6 versus temperature resembles the dielectric data ε´(T), having maxima corresponding to both dielectric anomalies at *T*_R_ and *T*_m_ (Figure 5f). Hence, the mechanism of reentrant transition for 6BZNb differs from that of BTZr and BZNb. There are still two competing ordering processes related to the system of BO_6_ octahedra: polar distortion and tilting, which however are in balance between *T*_R_ and *T*_m_. The maximum value of In7/In6 is ≈0.3 versus ≈0.8 for BTZr and 2BZNb, which indicates that the mesoscopic‐scale polar order within the B‐site sub‐lattice remains dominant over the tilt order. The minimum of ω_7_(T) at *T*_R_ further shows that the system of B‐site dipoles is stabilized below *T*_R_. Thus, the increase in entropy below *T*_R_ for 6BZNb should be related to the destabilization of the system of A‐site local dipoles, as revealed by the anomalous decrease of ω_1_(T) on cooling. One can speculate that for *x* = 0.06 the level of heterovalent substitution at the A‐site (Bi^3+^ for Ba^2+^) is sufficient to generate static random local electric and/or elastic fields below *T_R_*, which perturbs the order parameter developed at *T_m_*. It should be emphasized that for all three compounds BTZr, 2BZNb, and 6BZNb the FWHM Γ_1_ of the vibrational mode involving A‐site‐cation displacements is larger at 100 K than at 700 K. This reveals that for all the three compounds the degree of disorder within the A‐site sub‐lattice is larger in the low‐temperature reentrant phase (below *T_R_*) than in the high‐temperature phase (above *T_m_*). However, the temperature trends of ω_1_, ω_6_, ω_7_ and In_7_/In_6_ indicate that for BTZr and 2BZNb the A‐site sub‐lattice disorder below *T_R_* is indirect, caused by the evolving BO_6_ tilting and corresponding local elastic strains, in contrast to the case of 6BZNb.

**Figure 6:**
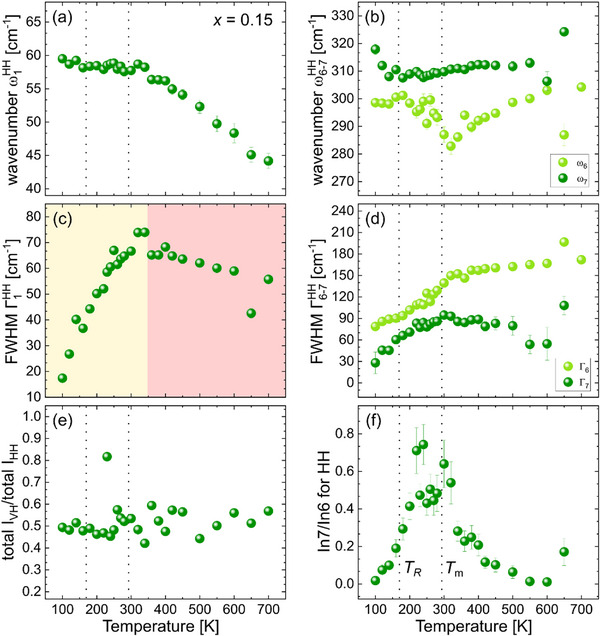
Temperature dependence of the Raman‐peak positions for ω_1_ (a), ω_6_, and ω_7_ (b), their corresponding FWHM (c, d) in the parallel‐polarized (HH) spectra, the intensity ratio of the cross‐polarized (VH) and parallel‐polarized (HH) spectra (e), and the fractional intensity ratio of the peaks ω_7_ and ω_6_ (f) for the BTZ‐BZNb sample with *x* = 0.15. The data points of ω_7_(T) below and above *T*_m_ are linearly fitted and their corresponding slopes are given in the plot (b).

**Figure** **6** displays the fitting results for the 15BZNb sample. In contrast to the other systems, it was not necessary to use a CP feature to fit the data at any temperature, which suggests no structural fluctuations or flipping of PNRs with a long relaxation time; the latter would narrow the CP so that its tail could not be detected above 15 cm^−1^. Thus, one can observe only two temperature regions in the trends for A‐site cation mode (Figure 6a,c) with a boundary at ≈350 K, which corresponds to *T*_B_ derived from dielectric data. The ω_6_(T) exhibits a minimum at 320 K (Figure 6c), which is close to *T*_m_, while Γ_6_(T) has a consistent maximum at *T*_m_. The depolarization ratio I_VH_/I_HH_ is constant in the entire temperature range (Figure 6e), revealing the absence of long‐range ferroelectric order. The temperature dependence of the intensity ratio In7/In6 exhibits a plateau‐like maximum between 220 and 320 K (Figure 6f), which matches well the plateau‐like trend of on *ε*´(*T*). No phonon softening or damping was observed at low temperatures (< *T*_m_), i.e., there is no evidence for the formation of a reentrant disordered state. Even ω_7_(T) stops to anomalously decrease on cooling, and near *T_R_*, as derived from *ε*´´(*T*), starts hardening i.e., no evolving instabilities related to BO_6_ tilting occur. Moreover, the FWHM Γ_1_ is considerably smaller at 100 K than at 700 K, indicating a lower degree of A‐site sub‐lattice disorder below *T_R_* than above *T_m_*.

## 3 Discussion

### 3.1 Correlation Between Dielectric Data and Raman Results

For all studied compositions, the dielectric permittivity shows both a frequency‐independent anomaly at high temperatures (*T_m_*) and a frequency‐dependent anomaly at lower temperatures (*T_R_*). This combination is often considered as a manifestation of the reentrant relaxor behavior, where the transition to a long‐range ordered ferroelectric‐like phase at T_m_ is followed by a reentrant relaxor phase below *T_R_*. Since the XRD patterns imply a pseudocubic structure for all compositions, the correlation length of polar distortions should be below the detection limit of XRD, and thus Raman spectroscopy helps to expose the microscopic mechanism behind the reentrant phenomenon.

Comparison between the dielectric and Raman spectroscopic results reveals anomalous behavior in the same temperature ranges for both data sets. Thus, a softening of the Raman mode related to the vibration of *B*‐cations within the oxygen octahedra (ω_6_) is observed at temperatures close to the maximum of the dielectric permittivity (*T_m_*) for all samples. This indicates the onset of correlated displacements of the B‐site cations. For the samples with *x* = 0 and 0.02 it is accompanied by an abrupt stepwise increase in the global intensity ratio *I_VH_/I_HH_* characteristic for the ferroelectric transition. Therefore, we can conclude that in these samples a long‐range ferroelectric order is established at *T_m_*. For compositions with larger concentration of BZNb, no stepwise increase in *I_VH_/I_HH_* is visible which suggests a further decrease in the correlation length and consequently, the size of ferroelectric domains. Importantly, no feature in the total intensity ratio *I_VH_/I_HH_* is observed around *T_R_*, even for the compositions with small *x*, which indicates that this dielectric anomaly is not related to a long‐range ordering.

The CP feature attributed to quasi‐elastic light scattering due to slow collective fluctuations of local dipoles can be seen for the samples with *x* ≤ 0.06. The onset of the CP feature upon cooling approximately matches with the Burns temperature derived from the dielectric data (Table 1), which can be explained by the formation of dynamic PNRs at *T_B_*.^[^ ^34^ ^]^ At temperatures below *T_R_*, the CP feature vanishes which suggest that the lifetime of polar fluctuations becomes long enough, i.e. the dynamics of the PNRs is frozen. The lack of the CP feature for the sample with *x* = 0.15 may indicate that PNRs in this composition are static or quasi‐static in the studied temperature range.

In BTZr, the dielectric anomaly at *T_R_* corresponds to a strong increase of the fractional intensity ratio of the Raman modes ω_6_ and ω_7_ (*In*_7_/*In*_6_) accompanied by anomalous softening of the ω_6_ mode. As discussed above, such behavior indicates a competition between the antiferrodistortive order related to correlated oxygen octahedra tilts and polar order due to collective displacements of the B‐site cations resulting in the destabilization of the latter. The compound with *x* = 0.02 also exhibits a stepwise increase in the *In*_7_/*In*_6_ ratio at *T_R_*. On the other hand, for the 6BZNb and 15BZNb samples the *In*_7_/*In*_6_ ratio is strong in the vicinity of *T_m_* but decreases below *T_R_* implying a decreased contribution of the correlated oxygen octahedra tilts at low temperatures.

### 3.2 Mechanism Behind the Reentrant Phenomenon

**Figure 7:**
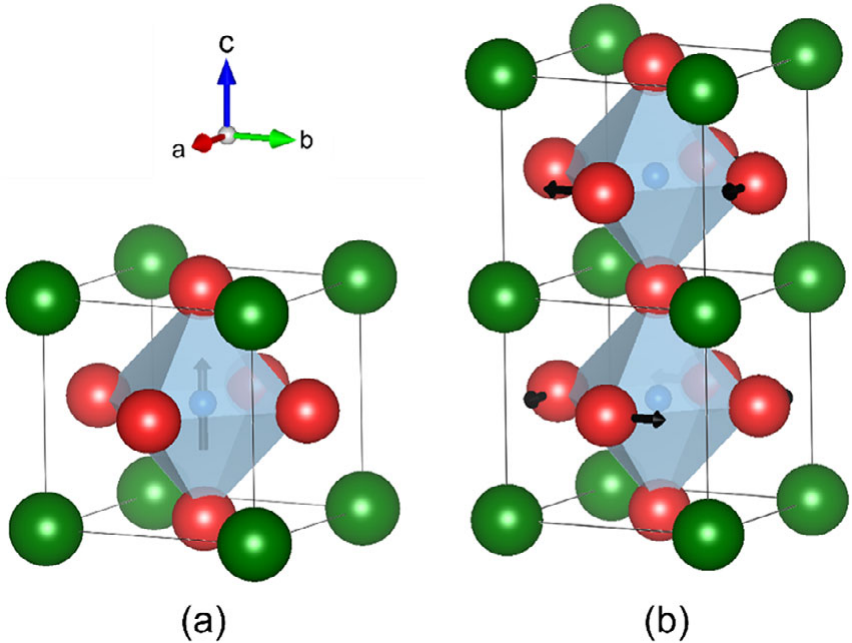
A sketch of competing order of polar displacement along the c‐axis in the ferroelectric macrodomains and the dynamic PNRs (a) and antiferrodistortive rotation of the oxygen octahedra around the c‐axis in the ”antipolar clusters” (b). The displacement and the rotation are indicated by black arrows.

Based on the presented findings from Raman spectroscopy, we suggest that the reentrant phenomenon in BTZr‐BZNb is related to structural instabilities and the coexistence of two competing ordering mechanisms. We propose that polar modes of the B‐cations compete with antiphase tilting of the oxygen octahedra (see **Figure** **7**). Both mechanisms dominate in different temperature ranges and are affected by the chemical substitution.

The competition between antiferrodistortive and ferroelectric instabilities was previously discussed for the model perovskite SrTiO_3_ by Aschauer and Spaldin.^[^ ^35^ ^]^ It was reported, that both components either compete or couple with each other, depending on the crystalline structure and the applied pressure.^[^ ^36^, ^37^, ^38^ ^]^ By applying tensile strain^[^ ^39^ ^]^ and partial cation doping,^[^ ^40^ ^]^ it is possible to overcome the suppression of the ferroelectricity in SrTiO_3_. Recent studies have shown that antiferrodistortive instabilities can also be found in BaZrO_3_.^[^ ^41^, ^42^, ^43^ ^]^ Levin *et al.* conducted a combined electron diffraction and total neutron scattering study of BaZrO_3_ and observed a structural change below 80 K related to correlated octahedral rotation in regions of 3 nm size.^[^ ^43^ ^]^ Using ab initio simulations, Sepliarsky *et al.* demonstrated that the replacement of Ti by Zr leads to intrinsic antiferrodistortive instabilities in the ABO_3_ structure with Ba at the A‐site, due to antiphase octahedral tilting.^[^ ^41^ ^]^ Based on the Raman spectroscopic data, we can assume that nanometer sized clusters of correlated octahedral tilts exist in all studied compositions. At high temperatures they are dynamic, but below *T_R_* these structural fluctuations freeze, forming static nanoclusters with antiphase tilts. The antiphase octahedral tilting promotes antipolar shifts of A‐site cations in double‐perovskite structural entities. In BTZr the large‐size A‐site Ba^2+^ cations have only weak off‐centered displacements and the corresponding contribution to the polarization and dielectric permittivity is small (Figure 1a). However, the substitution of Ba^2+^ by Bi^3+^, which has a strong tendency to off center due to its stereochemically active lone electron pair, increases the Raman signal at ω_1_ and significantly enhances the dielectric permittivity in the vicinity of *T_R_*. This speaks in favor of short‐range antipolar order of A‐site local dipoles, which coexists with the polar order of correlated displacement of B‐site (Ti^4+^) cations existing below *T_m_*. In the BTZr and 2BZNb samples, the stepwise increase in *I_VH_/I_HH_* at *T_m_* confirms the transition to the long‐range ferroelectric state, while in the latter composition a continuous softening of the ω_6_ mode between *T_m_* and *T_R_* points out frustration of the polar order most probably due to the competition with the antiphase BO_6_ tilts. For 6BZNb and 15BZNb the absence of an abrupt change in *I_VH_/I_HH_* indicates a decrease of the average size of spatial regions with correlated polar distortions, i.e. below *T_m_* there are static polar nanodomains rather than macroscopic domains as in the case of *x* = 0 and 0.02.

Thus, the reentrant relaxor behavior of BTZr‐xBZNb is attributed to the freezing of the dynamics of ferroic entities with short‐range antipolar order of the A‐site dipoles created by the correlated antiphase BO_6_ tilts. For easy of discussion, we refer to these entities as “antipolar clusters”. At high temperatures the structural fluctuations result in fluctuations of the dipole moments associated with these clusters. They possess their own dipole moments due to correlated B‐cation displacements as well as incomplete cancelation of dipoles related to the antipolar A‐cation displacements. The clusters are within the polar matrix of ordered Ti‐displacements, which can effectively mediate the interaction between them. We can assume that the polar order within the matrix is incoherent to the Ti‐displacements within the clusters.^[^ ^44^ ^]^ In this respect, the situation is different from the canonical relaxors where local polar nanoregions are embedded in a non‐polar but easy polarizable matrix.^[^ ^45^, ^46^, ^47^ ^]^ On cooling the interaction between the dipolar moments of the antipolar clusters leads to the slowing down of their dynamics and freezing below a certain temperature. It is important to mention that the characteristic timescale of Raman spectroscopy is ≈10^−12^ s, which is approximately the period atomic vibrations. Therefore, from the point view of atomic dynamics the freezing of the structural fluctuations at *T_R_*, means that they just become slow enough to generate a central‐peak tail, which could be detected in our Raman spectroscopic experiments. However, dielectric spectroscopy can measure the contribution related to the “flipping” of the dipole moments of the antipolar clusters on a much slower time scale. Therefore, the freezing temperature, *T_f_*, estimated from the Vogel‐Fulcher analysis of the dielectric data^[^ ^45^ ^]^ is smaller than *T_R_*.

Increasing the BZNb content has twofold consequences. On the one hand, Bi^3+^ substitution promotes off‐centering of A‐site cations leading to a strong dielectric relaxation at *T_R_*. On the other hand, the charge disorder on both A‐ and B‐sites progressively breaks the long‐range ferroelectric order of BTZr, which results in a suppression of the dielectric peak at *T_m_*. Most probably, the transformation from a long‐range macroscopic to a mesoscale polar order of the matrix is responsible for the abrupt changes in the activation energy and the attempt frequency of the low temperature relaxation (Table 1). In compositions with *x*  =  0 and 0.02 the values of *E_a_* and *f*_0_ are similar to those reported for canonical relaxors like PbMg_1/3_Nb_2/3_O_3_ with a strong interaction between polar nanoregions. One can assume that in this case the long‐range ordered polar matrix effectively mediates interaction between the antipolar clusters. On the other hand, for the compositions with short‐range static order (x = 0.06 and 0.15) the interaction between these clusters is weaker and the behavior is more like that of so‐called weakly‐coupled relaxors.^[^ ^48^ ^]^ While the dielectric permittivity data imply that BTZr‐15BZNb behaves like a reentrant relaxor similar to the composition with *x* = 0.06, the Raman data do not indicate any increase in structural disorder below *T_R_*. Therefore, we assume that in the case of 15BZNb the “antipolar clusters” are very slow already around *T_m_* (no detectable 0‐cm^−1^ feature) and besides, their fraction seems to be similar to the fraction of regions without additional ferroic order (similar level of *ε(T)* at *T_R_* and *T_m_*). Hence, the disorder in the state just below *T_m_* is already quite considerable to accommodate further increase in the entropy at *T_R_*.

The model of dynamic clusters embedded in an ordered polar matrix is similar to the random field model of the reentrant state. However, in our case the polar nature of the clusters and matrix are different. The former is related to the coexisting antipolar order of the A‐site dipoles and polar order of the B‐site dipoles, while the polar state of the matrix is determined by the coherent displacements of the B‐site‐cations.

## 4 Conclusion

BTZr‐BZNb ceramics exhibit a reentrant relaxor behavior in which a step‐like frequency dependent anomaly is observed in the temperature dependences of the dielectric permittivity below the ferroelectric phase transition. With increasing BZNb content, the peak permittivity corresponding to the ferroelectric phase transition decreases and becomes more diffuse, while the low‐temperature relaxor‐like anomaly becomes more pronounced. Based on the analysis of the dielectric and Raman spectroscopy data we attribute the reentrant relaxor behavior to the dynamics of nanometer size “antipolar clusters” within a polar matrix. The polar state of the matrix is due to correlated displacements of the B‐site cations. The increased disorder introduced by doping suppresses these correlations and leads to a transformation from the long‐range macroscopic to a mesoscale polar order. At the same time, antiferrodistortive tilts of the BO_6_ octahedra promote antipolar shifts of the A‐site cations. Since the BO_6_ tilts correlate only over a few nanometers, the ferroic entities comprising the A‐cation antipolar shifts act as faults within the polar matrix, increasing the entropy at lower temperatures. With increasing amount of the dopant, the interaction between the “antipolar” clusters weakens leading to a weakly coupled relaxor behavior. Our system demonstrates how the interplay between structural dynamics and functional properties in disordered systems, such as relaxor ferroelectrics, can result in unique behaviour driven by the coupling of local distortions.

## 5 Experimental Section

The BTZr‐BZNb ceramic samples were produced by conventional solid‐state method, with details available in Ref.[15] For the dielectric measurements, the pellets were polished, and electrodes were applied by silver pasting on the sample faces. To ensure the contact, the silver paste was thermally treated at 500 °C for 1 h. Dielectric spectroscopy was conducted between 37–500 K at frequencies from 100 Hz to 1 MHz using a HP 4284A precision LCR‐meter, combined with a Keithley Integra 2700 Multimeter and a Lakeshore 331 Temperature control.

For Raman spectroscopic experiments the samples were cut into pieces sized ≈2 × 2 × 0.5 mm^3^. Parallel (HH) and cross (VH) polarized spectra were collected in the range of 15–1215 cm^−1^ using a Horiba Jobin‐Yvon T64000 triple grating spectrometer equipped with an Olympus BX41 microscope. The spectra were excited using the 514.532‐nm wavelength of a Coherent Innova 90C FreD Ar^+^ laser. The instrumental accuracy in the peak positions was 0.35 cm^−1^, while the spectral resolution was 2 cm^−1^. The in situ temperature‐dependent spectra were collected on cooling from 700 to 100 K, using a Linkham600 heating/cooling stage purged with nitrogen. The temperature was measured with an accuracy of 0.1 K.

The as‐measured spectra were temperature‐corrected for the Bose‐Einstein population factor and then fitted with pseudo‐Voigt functions to obtain the phonon wave numbers ω, full width of half maximum Γ (FWHM), and integrated intensities *I*, following the data evaluation procedure described in.^[^ ^25^ ^]^ The fitting was conducted using the OriginPro 2023 software.

### Statistical Analysis

Raman measurements were performed on a representative sample, followed by detailed data evaluation. The raw spectra were corrected for temperature effects using the Bose‐Einstein population factor, then fitted with pseudo‐Voigt functions to extract the phonon frequencies (ω), full width at half maximum (Γ, FWHM), and integrated intensities (I). Curve fitting was carried out using OriginPro 2023.

At each temperature step, selected peaks were assessed using standard goodness‐of‐fit criteria: adjusted R^2^, reduced χ^2^, and the relative uncertainties of the fitted parameters. These criteria were also used to evaluate the statistical significance of individual fitting parameters. According to null‐hypothesis significance testing, a parameter is considered statistically significant if prob|t| < 0.05 (with a stricter threshold of < 0.005 used for higher confidence). If prob|t| exceeds 0.05 for a particular parameter, it is considered non‐significant and excluded from the model.

In particular, if the uncertainty in the integrated intensity (ΔI) of a peak exceeds half of its value (I), the associated prob|t| typically exceeds 0.05, and the peak is excluded from the fit. However, if metrics such as R^2^, reduced χ^2^, or visual inspection of the spectrum suggest the presence of an additional peak, all peaks are re‐evaluated by checking the relative errors of the integrated intensities and the corresponding prob|t| values.

Before modifying the number of peak functions in the fitting model, several alternative models are tested to ensure the robustness and reliability of the final results.

## Conflict of Interest

The authors declare no conflict of interest.

## Supporting information



Supporting Information

## Data Availability

The data that support the findings of this study are available from the corresponding author upon reasonable request.
