# Supplementary material for: Reentrant Phenomenon in Barium Titanate Zirconate‐Based Relaxor Ferroelectrics
Source: Small. 2025 Jul 15;21(35):2501914. doi: 10.1002/smll.202501914 (PMC12410908; doi:10.1002/smll.202501914)
Supplement: Supplementary file 1 — Supporting Information [file SMLL-21-2501914-s001.pdf]

NANO · MICRO
small

Supporting Information

for *Small*, DOI 10.1002/smll.202501914

Reentrant Phenomenon in Barium Titanate Zirconate-Based Relaxor Ferroelectrics

*Eva Kröll, Boriana Mihailova, Vadzim Haronin, Jūras Banys, Doru C. Lupascu and Vladimir V. Shvartsman**

Supporting Information

Reentrant Phenomenon in Barium Titanate Zirconate-Based Relaxor Ferroelectrics

*Eva Kröll, Boriana Mihailova, Vadzim Haronin, Jūras Banys, Doru C. Lupascu, and Vladimir V. Shvartsman**

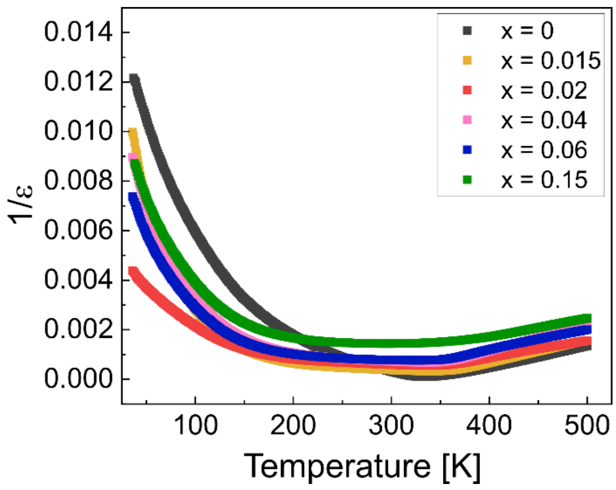

Figure S1. Reciprocal permittivity at 100 kHz of the BTZr-BZNb samples with x = 0, 0.015, 0.02, 0.04, 0.06, and 0.15 to determine T_B.

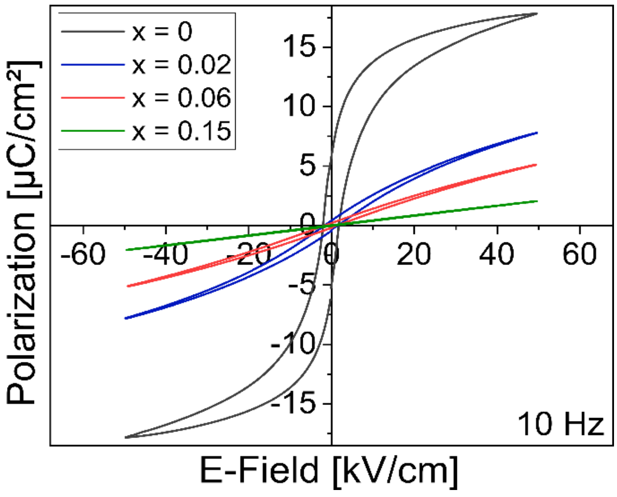

Figure S2. Polarization hysteresis loops of the BTZr-BZNb samples with x = 0, 0.02, 0.06, and 0.15 measured at room temperature.

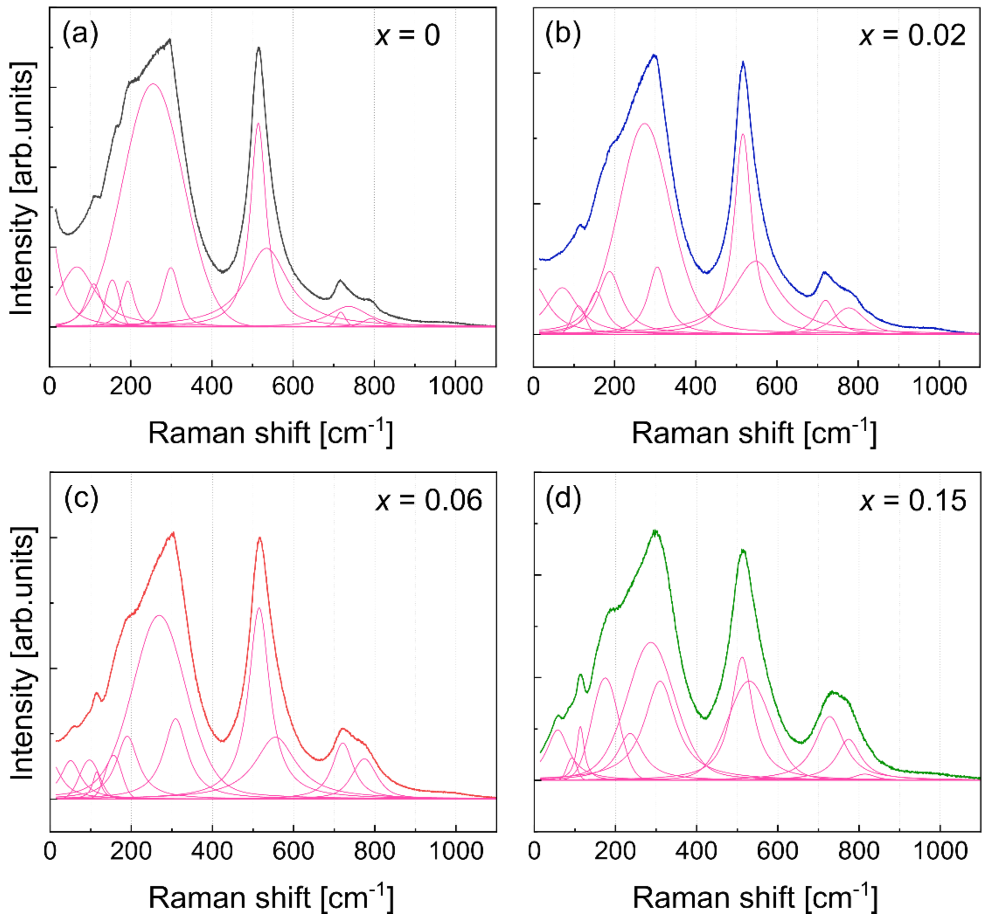

Figure S3. Raman spectra of the BTZr (a), 2BZNb (b), 6BZNb (c), and 15BZNb (d) samples measured at 300 K. The pink lines mark the Raman peaks that are discussed in this work.

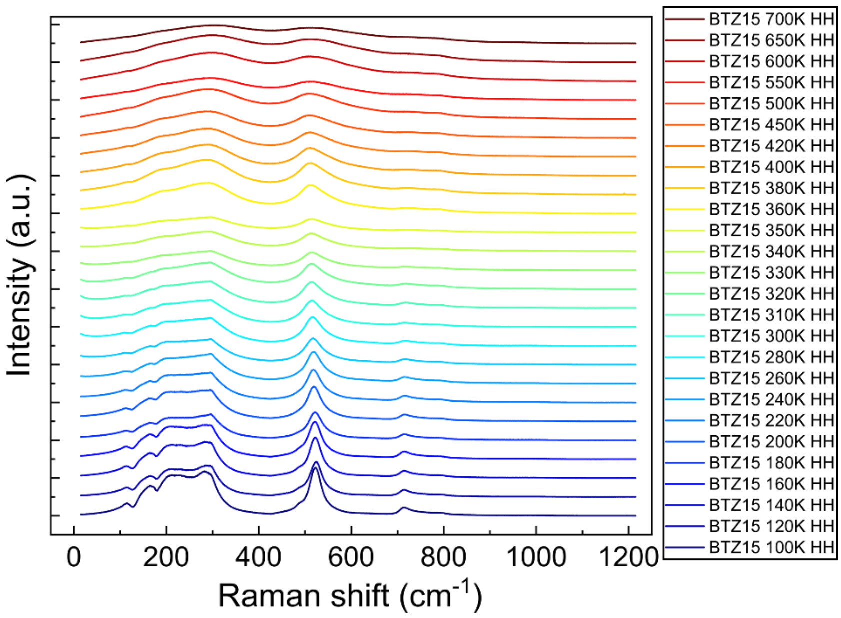

Figure S4. Temperature-dependent Raman spectra of the BTZr-BZNb sample with *x* = 0.

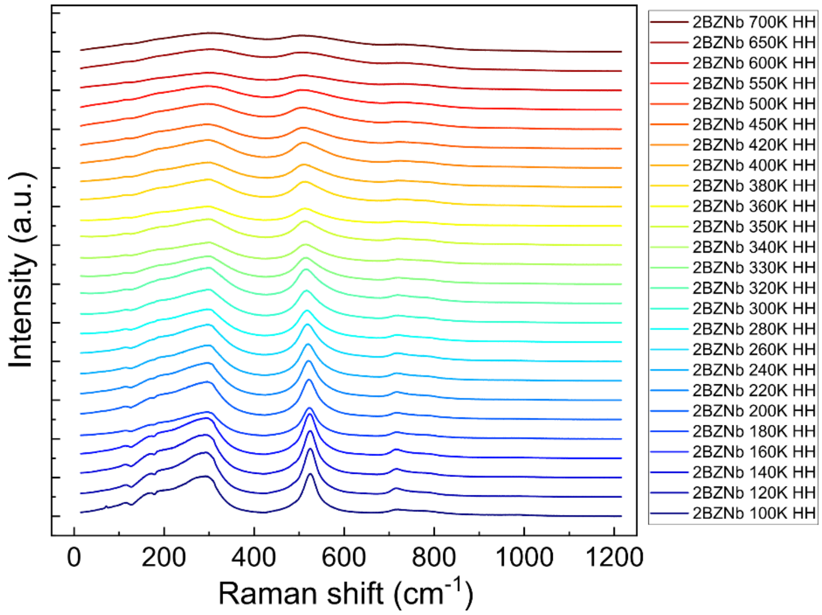

Figure S5. Temperature-dependent Raman spectra of the BTZr-BZNb sample with *x* = 0.02.

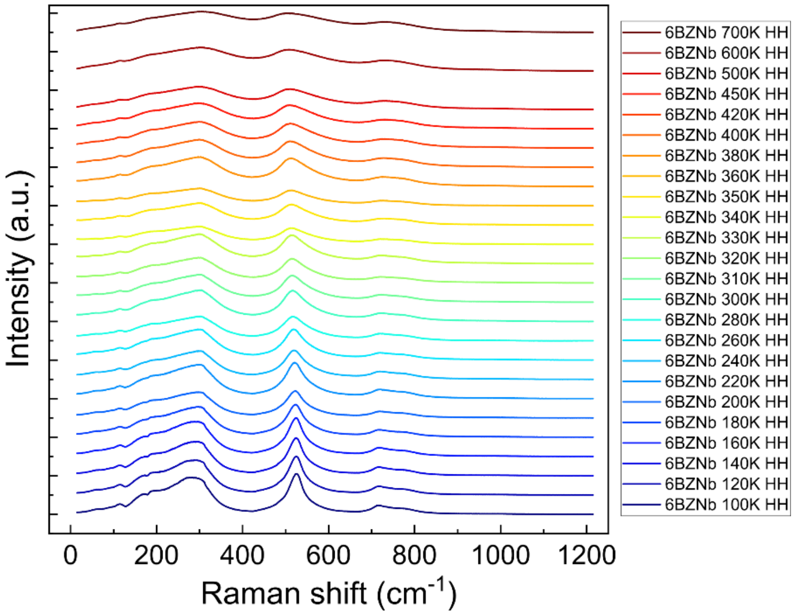

Figure S6. Temperature-dependent Raman spectra of the BTZr-BZNb sample with x = 0.06.

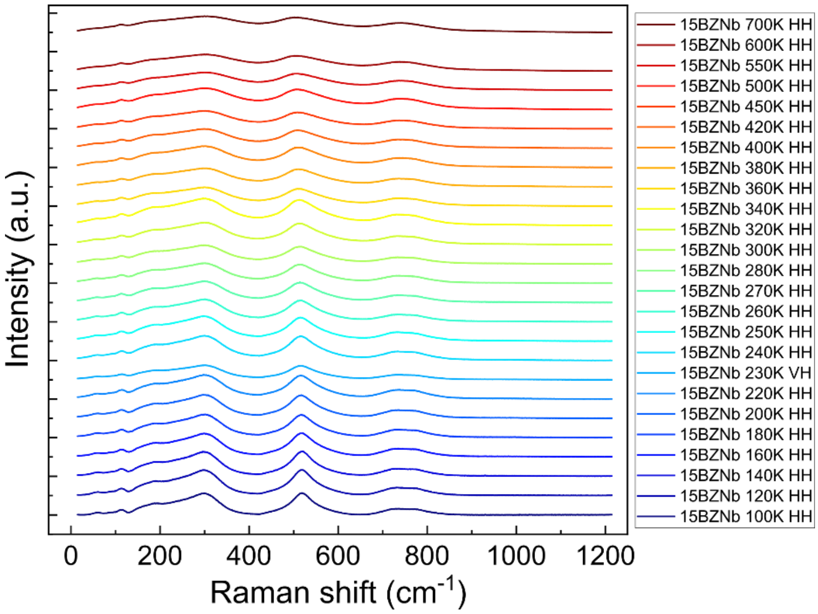

Figure S7. Temperature-dependent Raman spectra of the BTZr-BZNb sample with x = 0.15.
